# The effects of austerity measures on quality of healthcare services: a national survey of physicians in the public and private sectors in Portugal

**DOI:** 10.1186/s12960-017-0256-6

**Published:** 2017-12-12

**Authors:** Tiago Correia, Graça Carapinheiro, Helena Carvalho, José Manuel Silva, Gilles Dussault

**Affiliations:** 1 School of Sociology and Public Policies, ISCTE-Instituto Universitário de Lisboa, Av Forcas Armadas, 1649-026 Lisbon, Portugal; 2 Portuguese Medical Council, Av Almirante Gago Coutinho, 151, 1749-084 Lisbon, Portugal; 3 Global Health and Tropical Medicine, WHO Collaborating Center on Health Workforce Policy and Planning, Instituto de Higiene e Medicina Tropical, Universidade Nova de Lisboa, Rua da Junqueira, 100, 1349-008 Lisbon, Portugal

**Keywords:** Portugal, Economic crisis, Adjustment programs, Austerity measures, Quality of care, Trust

## Abstract

**Background:**

The European Union member countries reacted differently to the 2008 economic and financial crisis. However, few countries have monitored the outcomes of their policy responses, and there is therefore little evidence as to whether or not savings undermined the performance of health systems. We discuss the situation in Portugal, where a financial adjustment program was implemented between 2011 and 2014, and explore the views of health workers on the effects of austerity measures on quality of care delivery.

**Methods:**

A nationwide survey of physicians’ experiences was conducted in 2013–2014 (*n* = 3442). We used a two-step model to compare public and private services and look at the possible moderating effects of the physicians’ specialty and years of practice. Our data analysis included descriptive statistics, the independent *t* test, analysis of variance (ANOVA), multivariate logistic regression, General Linear Model Univariate Analysis, non-parametric methods (bootstrap), and post hoc probing.

**Results:**

Mainly in the public sector, the policy goal of maintaining quality of care was undermined by a lack of resources, the deterioration in medical residency conditions, and to a lesser extent, greater administrative interference in clinical decision-making. Differences in public and private services showed that the effects of the austerity measures were not the same throughout the health system. Our results also showed that physicians with similar years of practice and in the same medical specialty did not necessarily experience the same pressures.

**Conclusions:**

The debate on the effects of austerity measures should focus more closely on health workers’ concrete experiences, as they demonstrate the non-linearity between policy setting and expected outcomes. We also suggest that it is necessary to explore the interplay between lower quality and the undermining of trust relationships in health.

## Background

### Crises and health system performance: where do we stand in the debate?

How health services are planned, purchased, and delivered has a direct impact on the key dimensions of health system performance, i.e., efficiency, quality, and access [1]. In the aftermath of the economic and financial crisis that hit most member countries of the European Union, the question is raised of whether the impact of cuts in public funding and other austerity measures had neutral effects or have undermined the performance of health services [2].

As the crisis grew in intensity, the WHO Regional Office for Europe [3] recommended that member states monitor the effects of their policy responses on health indicators. Few countries did so, and existing assessments have focused mainly on measuring changes in access and efficiency, thus the need for further evidence on the dimension of quality of care delivery [4].

The analysis of health services delivery needs to take into account that the operation of provider organizations is more complex than assumed by political decision-makers [5]. This premise is based on the neo-institutional literature that argues that macro-level policies are affected by workplace-level contingencies that produce “perverse or non-expected” effects [6, 7].

This has received little attention in the debates on the effects on the quality of health services of political responses to the crisis. This paper is a contribution to the debate through a deeper look at how different levels of care, e.g., hospital and primary care, and the internal stratification of the medical profession, e.g., by specialty and years of service, constitute such contingencies [8–10]. That may explain why various categories of physicians, working in different environments, have reacted differently to the pressures generated by policy responses to the crisis.

Additionally, although public services have been the main target of austerity measures, complementarity with the private sector and growing pressure for marketization in many countries [11] raises the issue of understanding better how private providers have adapted to the economic crisis. There has not yet been systematic research into this matter even though some authors suggest that public regulation can be less effective in monitoring and penalizing for-profit investors for patient selection [12].

### The situation in Portugal

In 2011, a Memorandum of Understanding (MoU) between the Portuguese Government and the International Monetary Fund, The European Central Bank, and the European Commission designed a €78 billion, 3-year financial adjustment program with specific timelines and policy targets to reduce the budget deficit from 9.8 to 3% of GDP in 2013. Cost containment in the health sector was considered feasible without undermining the quality of services [13]. Direct cuts in the health sector were initially estimated at €550 million. However, they doubled after 1 year and reached €1.3 billion in 2013 [14].

Specific health-related policies were aimed at different targets: more cost-sharing, better drug-market regulation, tighter control over physicians’ prescribing and of the management of public provider organizations, more transparent public-private partnership, expansion of primary health care services, and savings on workforce costs. Table 1 shows the extent to which these objectives were achieved.

**Table 1:** Implementation of the MoU in Portugal 2011–2014

			Policy outcome			
			Accomplished	Partially accomplished	Not accomplished	Withdrawn/omitted
Cost sharing	Review and increase patient fees		x			
	Reduction of exemption categories		x			
	Increase inflation-indexed fees		x			
	Cut tax allowances for healthcare, including private insurance		x			
	Reduce the cost of health benefits schemes for public servants		x			
	Reduce costs for patient transportation		x			
Regulation of the drug market	Control retail price		x			
	Move the responsibility of pricing to the Ministry of Health		x			
	Revise the international reference-pricing system		x			
	Monitor expenditure monthly and limit public spending		x			
	Remove barriers to generic medicines		x			
	Change the calculation of pharmacies’ profit margin		x			
	Gradually increase the share of generic medicines		x			
	Implement existing legislation on the regulation of pharmacies					x
	Speed up the reimbursement of generics					x
	Introduce a contribution paid by pharmacies					x
Control of doctors’ prescription	Make electronic prescription of medicines and diagnostic tests covered by public reimbursement fully compulsory for physicians (public and private sectors)		x			
	Encourage physicians to prescribe generic medicines and less costly branded products (public and private sectors)		x			
	Introduce international prescription guidelines for drugs, exams and treatment		x			
	Improve monitoring of prescription of medicines and diagnostic services and impose systematic assessments by each doctor of quantity and cost. Introduce sanctions and penalties			x		
Control of operating costs and performance in the NHS	Legislative and administrative framework for a centralized procurement system for the purchase of medical goods		x			
	Change in the existing accounting framework in hospitals SOEs to that of private companies and other SOEs		x			
	Concentration and rationalization of non-hospital care provision		x			
	Concentration and rationalization of the hospital network		x			
	Continued publication of clinical guidelines and introduction of an auditing system		x			
	Benchmarking of hospital performance		x			
	Interoperability of IT systems in hospitals		x			
	Finalization and regular updates of uniform coding system for medical supplies			x		
	Implement the centralized purchasing of medical goods using the uniform coding system			x		
	Clearing of existing arrears in the hospital sector and prevention of accumulation of new arrears			x		
	Completion of patient electronic medical records				x	
Public-private relationship	Increase in competition between private providers and reduction in NHS payment of exams and treatments		x			
	Centralized monitoring of public-private partnership contracts		x			
	Regular revision of fees paid by the NHS for exams and treatment by private providers					x
	Assessment of compliance with European competition rules for the provision of services in the private healthcare sector					x
Access to healthcare	Reinforce primary health care	Increase the number of patients per primary care unit/family doctor	x			
		Increase the number of primary care units using salary and performance-related payments		x		
		Separate HR from hospitals and reconsider the role of nurses and other professionals			x	
		Review geographical distribution of GPs			x	
	Move hospital outpatient services to primary care units				x	
Workforce	Update working time, increase mobility, adopt flexible time arrangements and review payment mechanisms		x			
	Conduct an annual inventory of doctors		x			
	Make human resource allocation plans				x	
	Increase mobility of healthcare staff within and between regions				x	
	Ensure transparent selection of the chairs and members of hospital boards					x

Legend: Own elaboration from [26, 27]

Overall, the massive reduction in public expenditure came more from cost-sharing, tighter drug-market regulation, control over prescribing, and of the management of provider organizations. Improvements in public-private partnership, better access to primary healthcare, and workforce changes contributed to a lesser extent. Available studies of the effects of those reductions have focused mainly on efficiency and access [15] and planning and purchasing of services [16, 17].

### Objectives of the study

The aim of this research is to provide more evidence about the effects of an adjustment program on the quality of healthcare. Its contribution to the debate is threefold. First, quality needs to be considered as important as efficiency and access. Second, it highlights to what extent an analysis of delivery of healthcare requires looking at different sources of information than those on planning and purchasing of services. Health workers’ daily experiences are a rich source of information in this regard [18]. Third, it presents comparisons between and within the public and private sectors, a neglected aspect in research on policy responses to economic crises in Europe.

## Methods

### Study design and participants

**Fig. 1:**
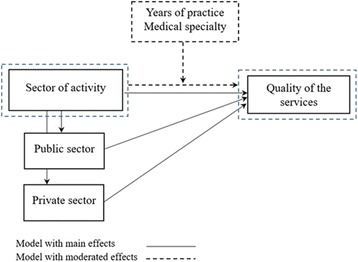
Analytical model

A two-step model (Fig. 1) was designed to describe the experiences of physicians working in public and private services (main-effect model) and analyze variations between the two groups based on years of experience and specialty (model with moderated effects). All types of public providers were included in the public sector, as were for-profit and professional-based insurers in the private sector.

Data was collected in a national survey asking physicians about their experiences after 2011, when the financial adjustment program began. A structured, self-administered questionnaire was sent by post from May 2013 to January 2014 to all physicians registered with the Portuguese Medical Council (*N* = 43,874). The key aim was to reach as many physicians as possible with no previous criteria underpinning the selection of cases. A total of 3442 questionnaires from physicians practicing in Portugal at that time were returned and validated through consistency and readability analysis. Even though it is one of the largest national-level databases of physicians in Europe, it was not possible to define a probabilistic sample of practicing physicians in Portugal because (i) there are no standardized data on the number of doctors who have retired or emigrated or on their distribution by sector or type of medical services and (ii) confidentiality criteria imposed by the Medical Council’s ethics committee prevented us from collecting the respondents’ age or geographical location.

### Measures

#### Dependent variables

Quality of care is analyzed in different ways depending on disciplinary backgrounds [19]. The analysis adopted here lay in a structure-process link, as it was intended to ascertain whether reforms changed how care was actually delivered [20]. Given that the questionnaire was sent to all registered physicians, we had to find suitable ways of comparing people in different positions and workplace settings. Therefore, the structure-process analysis was empirically driven according to the implementation of the MoU in Portugal from 2011 to 2014. Quality of care was addressed in terms of changes physicians said they had experienced in three key aspects of their daily practice: control of prescriptions, operating costs, and performance, as detailed in Table 1. Six dependent variables (DVs) were defined as follows:

-Administrative interference in decision making, i.e., less autonomy in physicians’ decision-making in favor of organizational control: more rejections of innovative treatments (DV1), pressure to choose cheaper treatments exerted by the administration (DV4), and pressure not to prescribe certain drugs exerted by the administration (DV5)
-Insufficient resources, i.e., possible limitation of material resources for the provision of care: regular shortages of supplies (gloves, masks, needles, etc.) (DV2) and regular shortages of drugs (DV3)
-Deterioration in medical residency, i.e., organizational changes that directly affected physicians’ advanced training: less favorable conditions for medical residencies (DV6) (e.g., overworked residents and less time for tutors’ work)

The list of questions was preceded by: “Based on your personal experience and in comparison to 2011”, and dichotomous dummy variables were defined with the answer “no” as a reference category (0). The proportion of “yes” answers was also used to make group comparisons.

#### Independent variables

The sector was analyzed at two levels: physicians working exclusively for the National Health Service (NHS) or exclusively in the private sector. The NHS included hospitals and primary health care and the private sector included small offices, clinics, and private hospitals.

#### Moderator variables

Years of practice meant the length of time physicians had been registered with the Medical Council (a compulsory requirement after post-graduate medical training). They were measured in months as a quantitative variable.

The 21 selected medical specialties (of a total of 41) were those of physicians working exclusively in the NHS or exclusively in the private sector. These were combined as follows: anesthesiology, cardiology, dermatology, gastroenterology, general practice/family medicine, general surgery, internal medicine, neurology, obstetrics and gynecology, oncology (medical and radiation oncology), ophthalmology, orthopedics, other surgical specialties (angiology and vascular, cardiothoracic, maxillofacial, pediatric surgery and plastic and reconstructive surgery, neurosurgery, and urology), ENT, pediatrics, physical medicine and rehabilitation, psychiatry, public health, pulmonology, radiology (nuclear medicine, neuroradiology, and radiology), and stomatology.

### Data analysis

Descriptive statistical analysis was used to examine the distribution of the variables, including non-answers. The independent *t* test and analysis of variance (ANOVA) were used to examine group differences on the basis of the proportion of “yes” answers for each dependent variable. A multivariate logistic regression was conducted to evaluate the moderator effect of years of practice and medical specialty in the relationship between sector of activity and quality of care using the Process Macro [21]. A General Linear Model (GLM) Univariate Analysis was conducted to assess the moderator effect of medical specialty testing interaction effects. Since some of the combinations of the factorial design showed a small number of cases, a non-parametric method (bootstrap) was also used to validate the results obtained by the parametric procedure. Some of the initial medical specialties had to be excluded in order to avoid empty combinations in the factorial design. Post hoc probing was conducted to interpret significant interactions utilizing estimates obtained from the fitted models [22] and also to achieve plotting interactions. All analyses were conducted using SPSS (version 23).

## Results

### Descriptive statistics

The final sample comprised 2063 physicians who had valid data according to the main inclusion criteria, working exclusively in the public or private sector (Table 2). Most respondents were general practitioners and working exclusively in the public sector. The percentage of younger doctors was slightly higher. As regards dependent variables, results were as follows: 17.3% reported more refusals of innovative treatments, 64.9% regular shortages of supplies, 30.6% regular shortages of drugs, 23.8% greater pressure to choose less costly treatments, 15.6% greater pressure to limit the prescription of certain drugs, and 48.9% less favorable conditions for medical residencies (Table 2).

**Table 2:** Descriptive statistics

Analyses variables and categories				*N*	%
Independent variable	Sector of activity	Exclusive in public		1209	58.6
		Exclusive in private		854	41.4
		Total		2063	100.0
		Exclusive in public	Primary healthcare	509	42.1
			Public hospitals	634	52.4
			Non answer	66	5.5
		Total		1209	100.0
		Exclusive in private	Small-size offices	274	32.1
			Clinics	230	26.9
			Hospital	79	9.3
			Non answer	271	31.7
		Total		854	100.0
Moderator variables	Years of practice	Up to 12 years		644	31.2
		13–25 years		473	22.9
		26–39 years		520	25.2
		Over 40 years		426	20.0
		Total		2063	100.0
	Medical specialty	Anesthesiology		59	2.9
		Cardiology		28	1.4
		Dermatology		17	.8
		Gastroenterology		23	1.1
		General practice/family medicine		498	24.1
		General surgery		110	5.3
		Internal medicine		132	6.4
		Neurology		16	.8
		Obstetrics and gynecology		78	3.8
		Oncology		38	1.8
		Ophthalmology		41	2.0
		Orthopedics		49	2.4
		Other surgical specialties		54	2.6
		Otorhinolaryngology		29	1.4
		Pediatrics		109	5.3
		Physical medicine and rehabilitation		26	1.3
		Psychiatry		64	3.1
		Public Health		28	1.4
		Pulmonology		31	1.5
		Radiology		36	1.7
		Stomatology		30	1.5
		Non answer		567	27.5
		Total		2063	100.0
Dependent variables—quality of care	DV1	No		1465	82.7
		Yes		307	17.3
		Total		1772	100.0
	DV2	No		639	35.1
		Yes		1184	64.9
		Total		1823	100.0
	DV3	No		1149	69.4
		Yes		506	30.6
		Total		1655	100.0
	DV4	No		1384	76.2
		Yes		432	23.8
		Total		1816	100.0
	DV5	No		1819	84.4
		Yes		336	15.6
		Total		2155	100.0
	DV6	No		747	51.1
		Yes		716	48.9
		Total		1463	100.0

Notes: (1) Sums of subject numbers for the dependent variables are not always equal because of missing data; percentages are based on number of subjects for whom data were available; (2) DV1—refusal of innovative treatments; DV2—regular shortage of work supplies; DV3—shortage of drugs; DV4—pressure to choose less-expensive treatments; DV5—pressure not to prescribe specific drugs; DV6—inferior medical training

### Group comparisons

A comparison of means between sectors showed that they significantly affected physicians’ evaluation of quality of care in several indicators: usual shortage of supplies (*t* (1054) = 14.379, *p* < .001) (DV2), shortage of drugs (*t* (938) = 4.809, *p* < .001) (DV3), pressure to choose cheaper treatments (*t* (1049) = 3.001, *p* = .003) (DV4), pressure to limit the prescription of certain drugs (*t* (1276) = 6.044, *p* < .001) (DV5), and less favorable conditions for medical residencies (*t* (684) = − 2.439, *p* = .015) (DV6). Except for the latter, there were more “yes” answers among physicians working exclusively in the public sector (see 1st step, Table 3).

**Table 3:** Comparisons of quality of care between groups (t-test and one-way ANOVA)

Sector of activity		DV1	DV2	DV3	DV4	DV5	DV6
		Mean^a^	Mean	Mean	Mean	Mean	Mean
1st step							
Public		0.153	0.731	0.300	0.262	0.200	0.435
Private		0.134	0.228	0.110	0.158	0.058	0.571
Model test		*t* (1026) = 0.683	*t* (1054) = 14.379***	*t* (938) = 4.809***	*t* (1049) = 3.001**	*t* (1276) = 6.044***	*t* (684) = − 2.436*
2nd step							
Public	Primary healthcare	0.065	0.847	0.091	0.351	0.267	0.385
	Public hospitals	0.234	0.637	0.437	0.201	0.143	0.471
Model test		*t* (786) = − 6.663***	*t* (815) = 6.893***	*t* (751) = − 10.914***	*t* (820) = 4.869***	*t* (910) = 4.741***	*t* (554) = − 1.996*
Private	Small-size offices	0.286	0.125	0.100	0.161	0.056	0.636
	Clinics	0.074	0.333	0.129	0.239	0.061	0.308
	Hospital	0.167	0.188	0.133	0.194	0.100	0.750
Model test		*F* (2, 116) = 3.682*	*F* (2, 115) = 2.768	*F* (2, 78) = 0.0660	*F* (2, 105) = 1.609	*F* (2, 201) = 0.514	*F* (2, 48) = 3.299*

^a^Mean = proportion of answer “yes”

*p < .05; **p < .01; ***p < .001

Significant differences were found among physicians working exclusively at public hospitals and in primary care in all dependent variables (*p* < .05). Physicians working exclusively in public hospitals mentioned refusal of innovative treatments (*t* (786) = − 6.663, *p* < .001) (DV1), drug shortages (*t* (751) = − 10.914, *p* < .001) (DV3) and, with a smaller difference, less favorable conditions for medical residencies (*t* (554) = − 1.996, *p* = .023) (DV6). Physicians working in primary care services mentioned shortages of equipment (*t* (815) = 6.893, *p* < .001) (DV2), pressure to choose cheaper treatments (*t* (820) = 4.869, *p* < .001) (DV4), and pressure not to prescribe certain drugs (*t* (910) = 4.741, *p* < .001) (DV5).

Significant differences were found among physicians working in the private sector only regarding refusal of innovative treatments (*F* (2, 116) = 3.682, *p* = .028) (DV1) and, to a lesser extent, less favorable conditions for medical residencies (*F* (2, 48) = 3.299, *p* = .045) (DV6). There were fewer “yes” answers in both indicators among physicians working in clinics (see 1nd step, Table 3).

### Moderator analysis

A moderator effect of years of practice was tested to provide a more detailed understanding of the differences between working in the public or private sector (Fig. 1). The interaction effect between sector and years of practice had a significant effect on refusal of innovative treatments (*B =* − 0.005, *Z =* − 2.396, *p* = .017, 95% CI = − 0.008, − 0.001) (DV1), pressure not to prescribe certain drugs (*B =* 0.005, *Z =* 2.884, *p* = .004, 95% CI = 0.002, 0.009) (DV5) and less favorable conditions for medical residencies (*B =* − 0.004, *Z =* − 2.075, *p* = .038, 95% CI = − 0.007, − 0.001) (DV6) (Table 4).

**Table 4:** Multivariate logistic regression model with the moderator effect of years of practice on quality of care

	DV1	DV2	DV3	DV4	DV5	DV6
Exclusive in public^a^	1.556	1.160^*^	− 0.104	− 0.115	− 0.814	0.571
	(0.857)	(0.580)	(0.714)	(0.640)	(0.710)	(0.692)
Years of practice (exclusive in private)	0.002	− 0.003^**^	− 0.005^**^	− 0.002	− 0.006^**^	0.001
	(0.002)	(0.001)	(0.002)	(0.002)	(0.002)	(0.002)
Exclusive in public * Years of practice^b^	− 0.005^*^	0.002	0.002	0.002	0.005^**^	− 0.004^*^
	(0.002)	0.001	(0.002)	(0.002)	(0.002)	(0.002)
Constant	− 2.742^**^	0.212	− 0.229	− 0.775	− 0.522	0.172
	(0.843)	(0.563)	(0.703)	(0.626)	(0.694)	(0.645)
Model LL =	18.316^***^	186.426^***^	57.636^***^	13.628^**^	52.207^***^	24.265^***^
Post hoc tests						
Years of practice (exclusive in public)	− 0.003^***^	− 0.002^**^	− 0.003^***^	− 0.002^**^	− 0.002	0.000
	(0.001)	(0.001)	(0.002)	(0.001)	(0.001)	(0.002)

Non-standardized coefficients and standard errors (in brackets) are reported

^a^Exclusive private (baseline)

^b^Interaction effect

*p < .05; **p < .01; ***p < .001

**Fig. 2:**
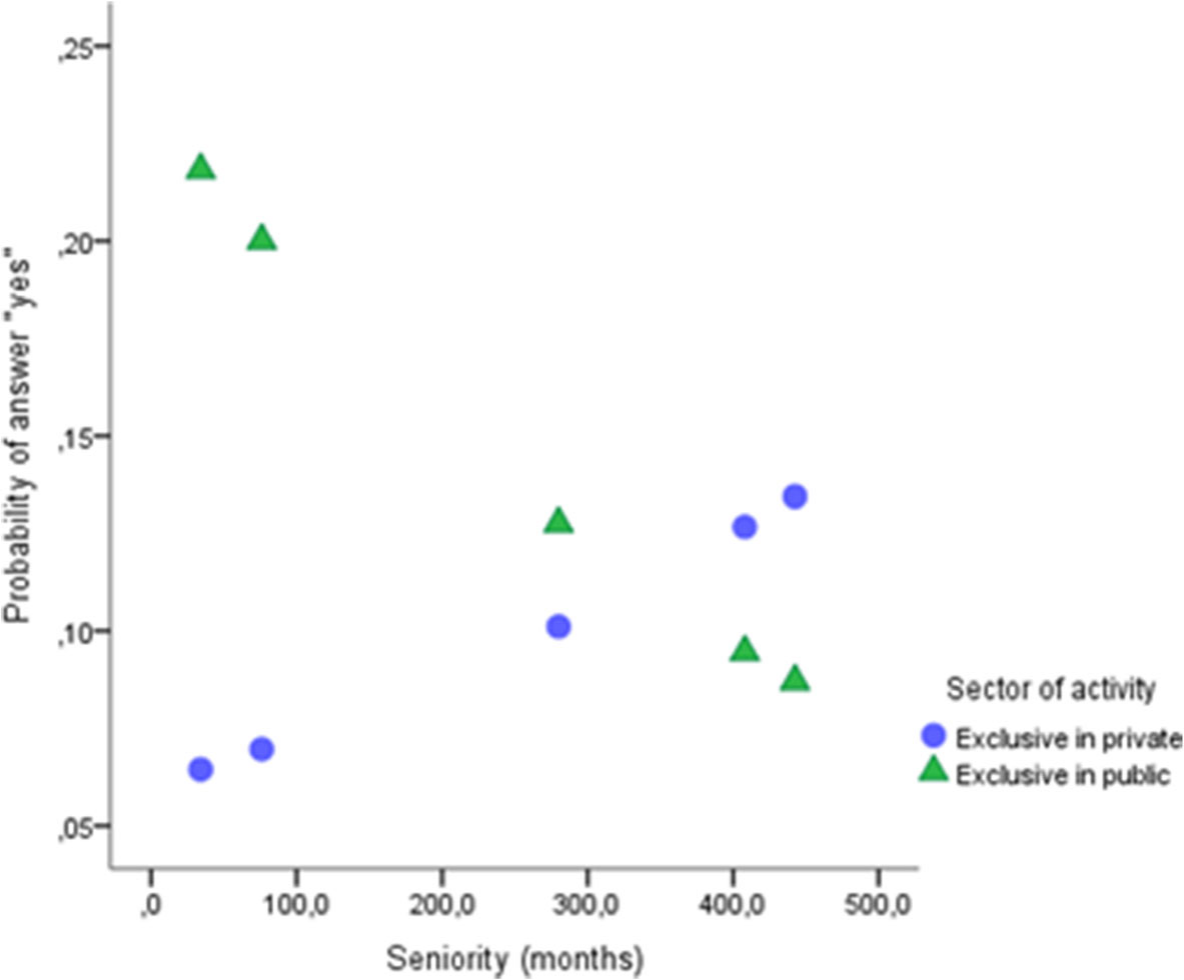
Interaction between sector and years of practice in refusal of innovative treatments

Figure 2 shows that refusal of innovative treatments tended to be reported more by older physicians in the private sector and younger physicians in the public sector. Post hoc tests revealed a significant effect of years of practice only in the public sector (*B =* − 0.003, *Z =* − 3.941, *p* = .000, 95% CI = − 0.004, − 0.001).

**Fig. 3:**
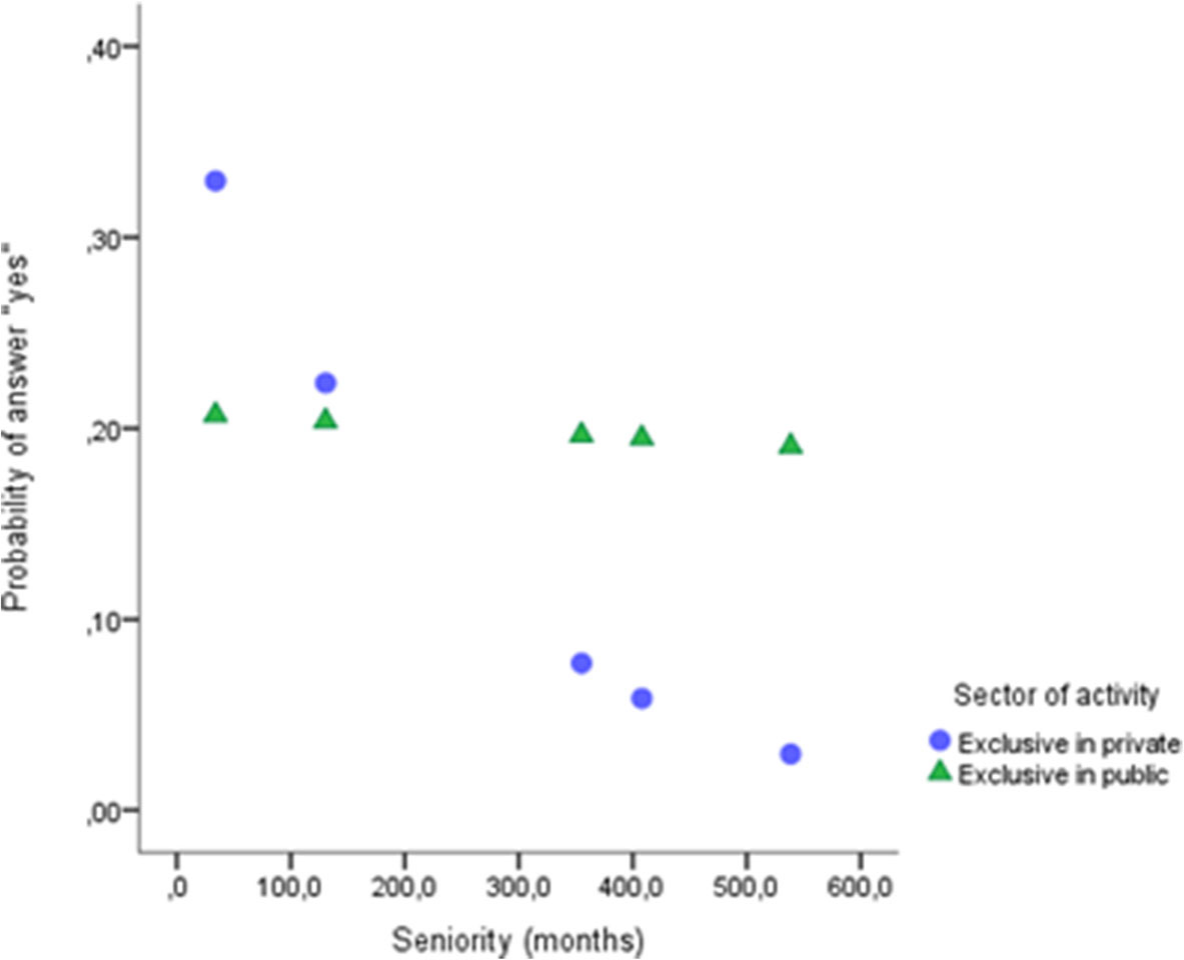
Interaction between sector and years of practice in pressure not to prescribe certain drugs

As presented in Fig. 3, the pressure not to prescribe certain drugs was not affected by years of practice in the public sector (*p* > .05). A significant negative effect was found in the private sector and was less likely to occur among older physicians (*B =* − 0.006, *Z =* − 3.121, *p* = .002, 95% CI = − 0.009, − 0.002) (Fig. 3).

**Fig. 4:**
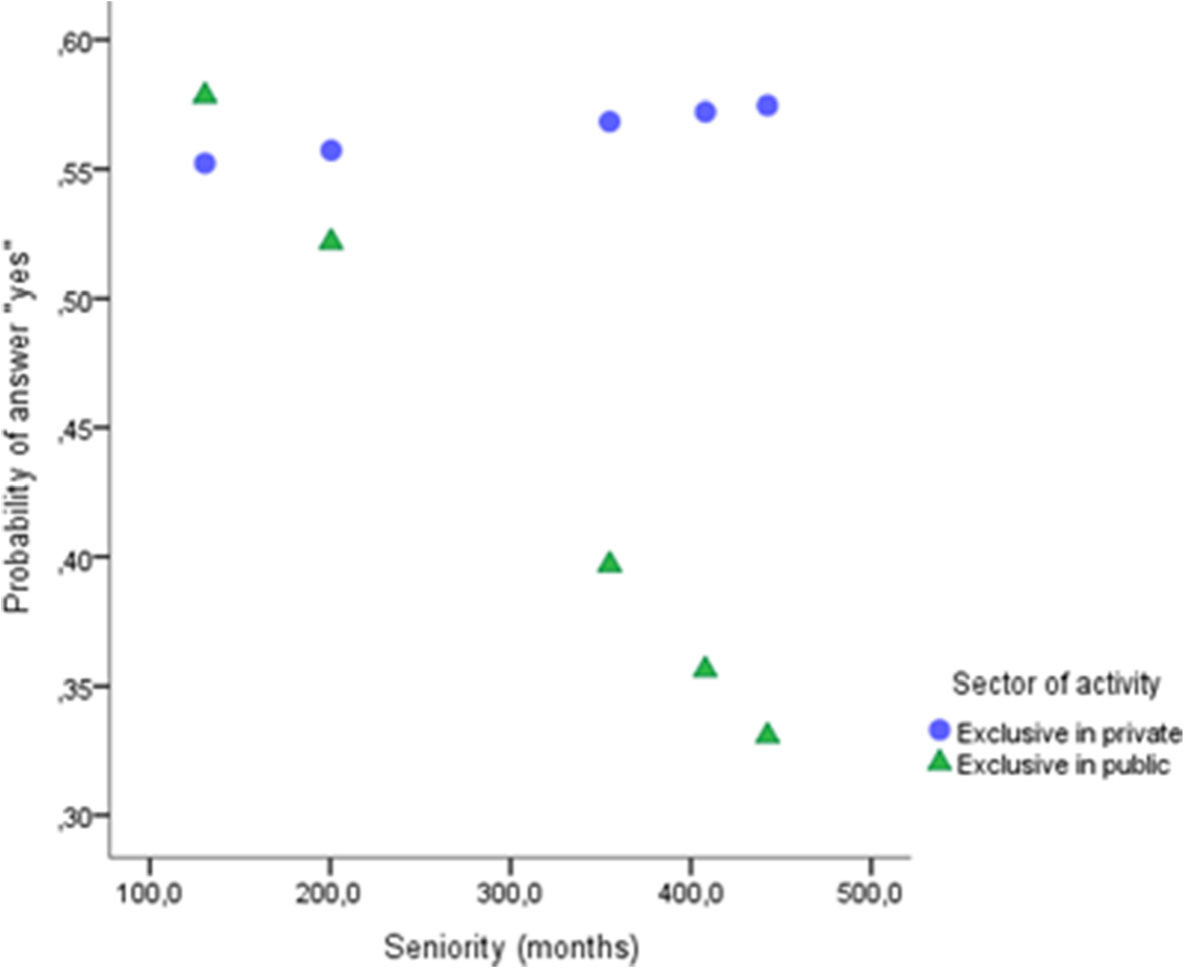
Interaction between sector and years of practice in deterioration in medical training

In contrast, less favorable conditions for medical residencies were not affected by years of practice in the private sector (*p* > .05), while a significant negative effect was observed in the public sector (*B =* − 0.003, *Z =* − 4.219, *p* < .001, 95% CI = − 0.005, − 0.002) (Fig. 4).

Regarding regular shortages of supplies and drugs, only the main effect was significant, as years of practice did not have a moderating effect (*p* < .05) (Table 4). Older physicians made less mention of regular shortages of supplies in both the private and public sectors (*B =* − 0.003, *Z =* − 2.594, *p* = .01, 95% CI = − 0.006, − 0.001 and *B =* − 0.002, *Z =* − 3.238, *p* = .001, 95% CI = − 0.003, − 0.001, respectively). Drug shortages also produced fewer “yes” answers from older physicians and this significant effect occurred in both sectors (*B =* − 0.005, *Z =* − 2.622, *p* = .009, 95% CI = − 0.008, − 0.001 and *B =* − 0.003, *Z =* − 4.821, *p* < .001, 95% CI = − 0.004, − 0.002, private and public respectively).

Moderation by medical specialty was also tested (Fig. 1). The interaction between sector and medical specialty had a significant effect on refusal of innovative treatments (*F* (11, 595) = 1.988, *p* = .035) (DV1) and shortages of drugs (*F* (11, 520) = 4.206, *p* < .001) (DV3) (Table 5).

**Table 5:** GLM Univariate Analysis with the moderator effect on the relationship between medical specialty and quality of care

	DV1	DV2	DV3	DV4	DV5	DV6
Sector	*F* (1, 595) = 0.842	*F* (1, 609) = 68.809***	*F* (1, 520) = 23.010***	*F* (1, 606) = 0.006	*F* (1, 764) = 0.571	*F* (1, 382) = 2.038
Medical specialty	*F* (11, 595) = 2.701**	*F* (11, 609) = 3.447***	*F* (11, 520) = 0.898	*F* (11, 606) = 1.130	*F* (11, 764) = 1.143	*F* (11, 382) = 0.927
Interaction between sector and medical specialty	*F* (11, 595) = 1.988*	*F* (11, 609) = 1.370	*F* (11, 520) = 4.206***	*F* (11, 606) = 0.993	*F* (11, 764) = 0.822	*F* (11, 382) = 1.353

*GLM* General Linear Model

*p < .05; **p < .01; ***p < .001

**Fig. 5:**
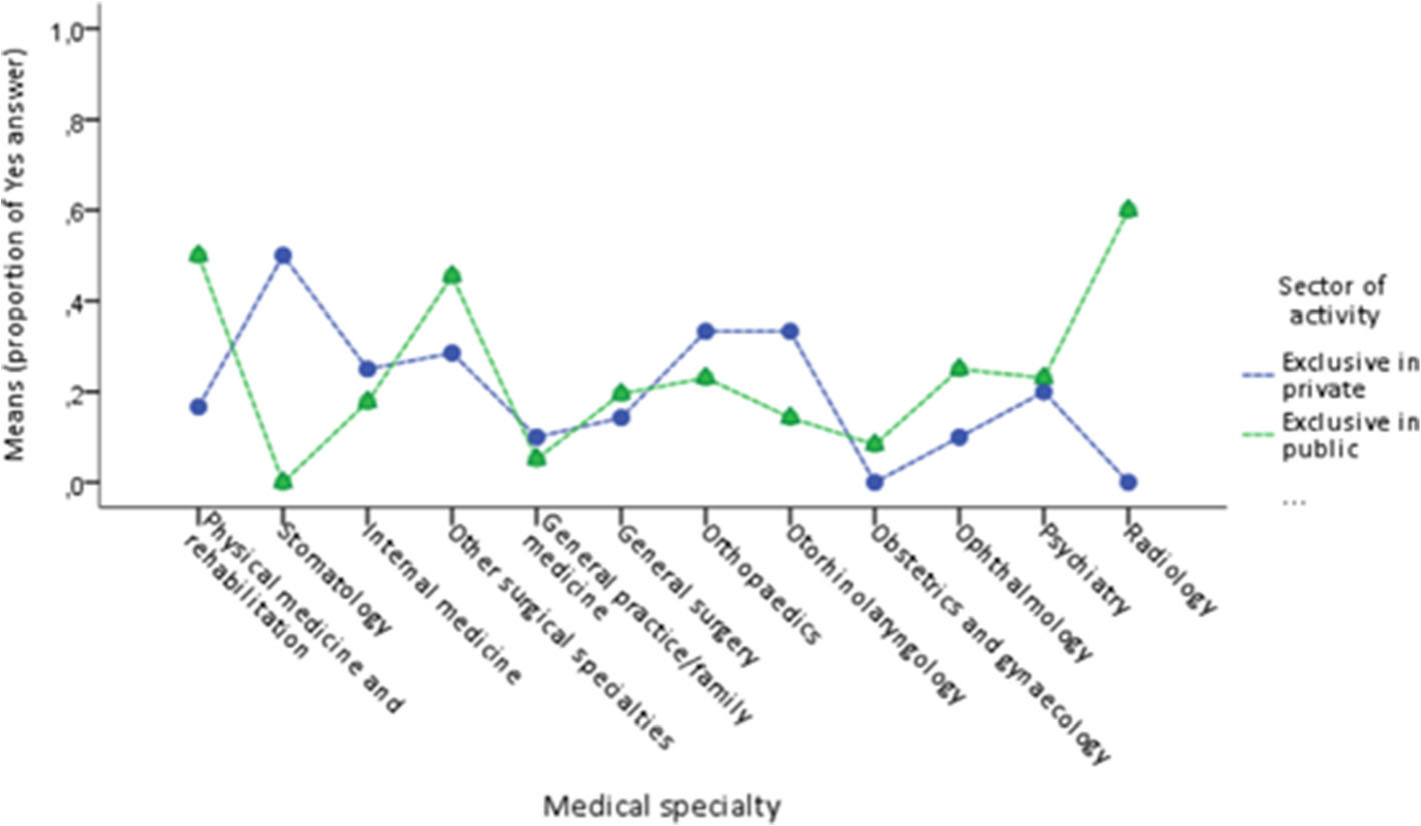
Interaction between sector and medical specialty in refusal of innovative treatments

**Fig. 6:**
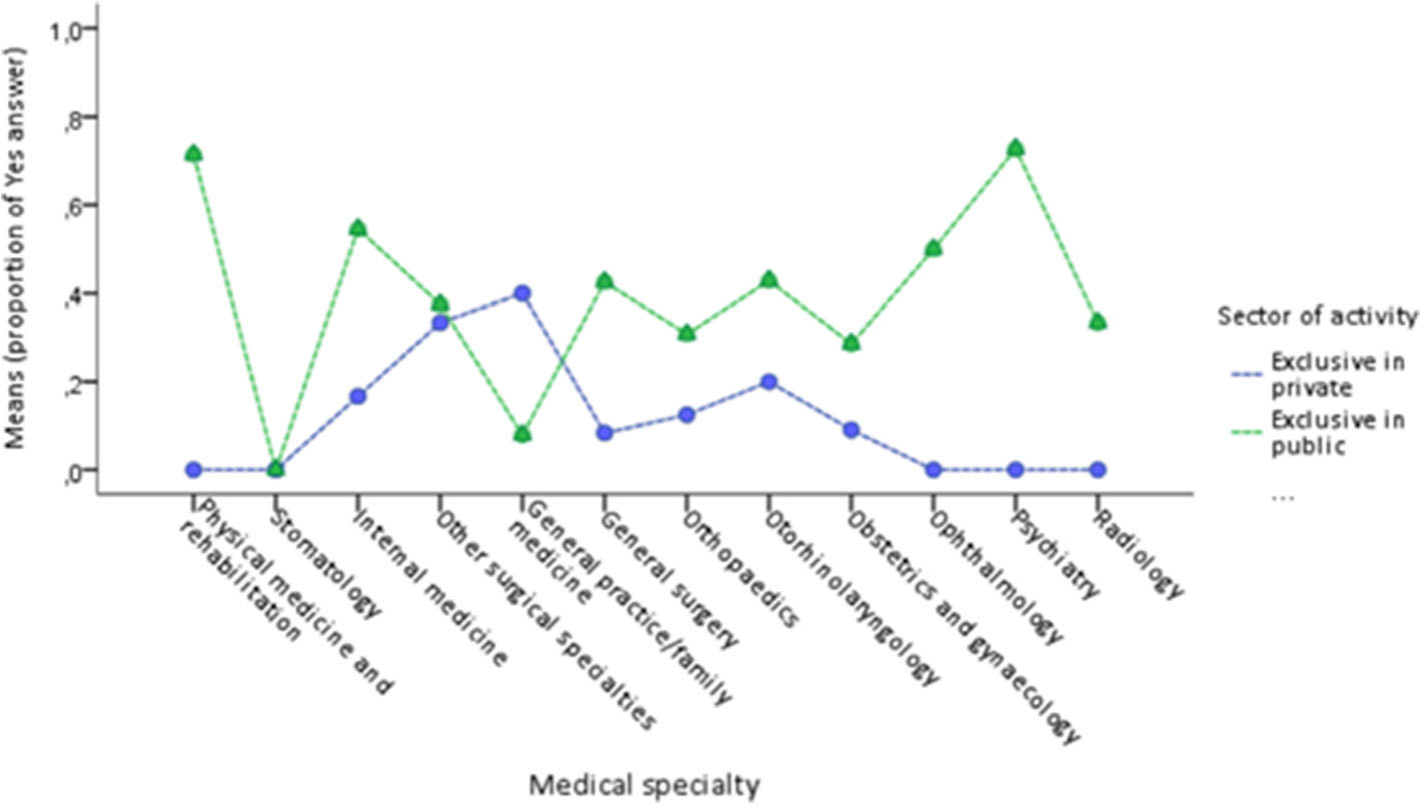
Interaction between sector activity and medical specialty on shortage of drugs

There was a significant difference between the two sectors for refusal of innovative treatments in stomatology (*p* = .024) and radiology (*p* = .004). On the other hand, the answer “yes” was more frequent for oral medicine in the private sector and radiology in the public sector (Fig. 5). Shortages of drugs were significantly different between the sectors in various specialties: physical medicine and rehabilitation (*p* = .001), internal medicine (*p* = .020), general practice/family medicine (*p* = .020), general surgery (*p* = .006), ophthalmology (*p* = .038), and psychiatry (*p* < .001). With the exception of general practice/family medicine, the answer “yes” was more frequent in the public sector for all the other medical specialties (Fig. 6).

## Discussion

The understanding of the effects of adjustment programs like those of Greece, Portugal, Ireland, and Cyprus fills an important gap in the literature on policy responses in Europe to the recent economic crisis. Conclusions result in non-convergent evidence and therefore further analysis is required. The framework for this study was built on two overlooked assumptions. First, the understanding of the effects of the crisis on health systems performance is still limited given that few analyses have been conducted on quality of care as compared to efficiency and access. Second, looking at healthcare delivery is likely to reveal differences in relation to the analysis of planning or purchasing due to workplace-level contingencies. This led us to assess, based on the experience of those who provide care, whether structural reforms impacted the care delivery process and the quality of services.

Accordingly, we suggest that the reforms made under the Portuguese adjustment program had negative effects on the quality of care, namely in terms of shortages of work resources, less favorable conditions for medical residencies, and to a lesser extent, more administrative interference in clinical decisions. Reports of negative effects were consistently higher among physicians working exclusively for the NHS, which illustrates that the public sector was the main target of health-related austerity measures, even though these were expected to apply to all types of providers without exception.

Our findings show also that the austerity measures were felt differently in the public and private sectors. Results in the NHS suggest insufficient resources and greater constraints on physicians’ individual decision-making (e.g., equipment shortages and administrative interference in clinical decisions). In the private sector, the main outcome of reforms was the negative impact on medical residencies due to overworked residents and to less time for tutors to provide training. Constraints in both sectors require further studies for a better understanding of its effects on clinical autonomy and training and ultimately on health outcomes.

Looking at public and private services, physicians in public hospitals reported more denials of innovative treatments, more drug shortages, and less favorable conditions for medical residencies. Primary care physicians reported more equipment shortages and administrative interference in medical decisions. In the private sector, physicians in small offices reported more refusals of innovative treatments than those in clinics and private hospitals, while the latter complained more deterioration in medical residency.

The argument that physicians’ experiences depend on the sector of activity is reinforced when we analyzed the moderating effects of two key factors in medical hierarchy (years of practice and specialty). The reporting of denial of innovative treatments decreases with seniority in the public sector, while it increases in the private sector. Administrative interference was perceived as higher by less-experienced physicians in the private sector, while in the public sector years of service made no difference as to the pressure to limit the prescription of certain drugs. More less-experienced physicians in the public sector mentioned less favorable conditions for medical residencies, while this trend was not found in the private sector. As to differences between medical specialties, refusal of innovative treatments was more reported by stomatologists in the private sector than those in the public sector and by radiologists in the public sector than those in the private sector. Shortage of drugs was more reported by public-sector physiologists, internists, surgeons, ophthalmologists, and psychiatrists than by these specialists in the private sector, while general practitioners in the private sector reported it more than their colleagues in the NHS.

Our study shows that physicians in public and private sectors and within each sector have different perceptions, depending on their specific characteristics, of the impact of the same policy responses.

A number of potential long-term effects that this empirical study cannot address are associated with these results. One in particular is here uncovered to highlight possible directions for research. Lower quality healthcare services presumably affect health professionals’ and patients’ individual choices. Given that the influence of trust in individuals’ decisions is well understood [23, 24], the understudied link is the effects of lower quality on the undermining of trust relationships in health. The issue is to know how lower quality affect professionals’ and patients’ individual choices and whether these choices can affect health systems structurally.

As to the NHS, the quality-trust link builds on the moral contract between authorities, citizens, and professionals. Professionals feeling that they are not being given the conditions they need for their work and patients questioning the need for higher taxation to fund access to services that are less trustworthy can translate into the growth of the private market in parallel to the public sector. As to the private sector, the quality-trust link is a key element in market competition. Greater pressure is put on the supply side in investment in communication strategies and patient-centered care approaches. Consequently, it is relevant to discuss possible increases in households’ spending and growing exposure to deregulation of the health labor market.

### Limitations

We were unable to establish the representativeness of the respondents, even though the total number of over 3000 is high. Also, the instrument does not permit a clear assessment of the moderating effect of physicians’ years of practice and specialties, selected as proxy of internal stratification of the medical profession. More empirical in-depth research into this issue is needed. Lastly, the measurements of quality of care delivery reflect physicians’ personal experiences and views and are therefore not comparable to those of health workers.

## Conclusion

The aim of this article was to add to the understanding of the effects of policy responses to the 2008 crisis on health system performance in Europe. In Portugal, there is an institutional discourse that the health system adapted well to cost containment [25]. By focusing on the perceptions of professionals of the effects of the crisis on their work and on the quality of care, we offer a complementary contribution. Also, our findings show that the outcomes of policy responses may not have been the same in all national health care systems, therefore requiring context-sensitive analyses.

The added value of an analysis built on a macro (political)-micro (organizational) link is likely to reveal a different reality from that showed by general statistics, which do not reflect concrete experiences. It also shows the non-linearity between policy setting and expected outcomes, which is particularly relevant in assessing the effects of austerity measures. The fact that only physicians were surveyed and the lack of standardized data on physicians in Portugal are limitations that further research can help mitigate.

## Abbreviations

DV: Dependent variable
MoU: Memorandum of Understanding
NHS: National Health Service
